# The community capacity curve applied to reforestation: a framework to support success

**DOI:** 10.1098/rstb.2021.0079

**Published:** 2023-01-02

**Authors:** John Herbohn, Liz Ota, Nestor Gregorio, Robin Chazdon, Robert Fisher, Jack Baynes, Grahame Applegate, Tony Page, Dora Carias, Claudia Romero, Francis E. Putz, Jennifer Firn

**Affiliations:** ^1^ Tropical Forest and People Research Centre, University of the Sunshine Coast, 90 Sippy Downs Drive, Sippy Downs, QLD 4556, Australia; ^2^ School of Geosciences, University of Sydney, NSW, Australia; ^3^ Department of Biology, University of Florida, Gainesville, FL 32611-8526, USA; ^4^ School of Biological and Environmental Science, Queensland University of Technology, Brisbane 4001, Australia

**Keywords:** forest and landscape restoration, sustainable livelihoods framework, community forestry, forest restoration, Community Capitals Framework

## Abstract

Community involvement is critical for the success of many interventions designed to promote reforestation. To secure this involvement, it helps to recognize that communities are heterogenous both within and among themselves and possess diverse mixes of livelihood assets required to implement reforestation. We explore the relationship between livelihood assets and reforestation success and outline a conceptual model that we call the community capacity curve (CCC) applied to reforestation. We argue that the shape of the CCC is sigmoidal. Importantly, communities at the lower end of the CCC have limited capacity to implement reforestation projects without substantial and ongoing capacity building and other sorts of support, including through livelihood projects that improve food security and provide cash benefits. Communities at the higher part of the CCC have greater capacity to implement reforestation projects, especially projects focused on biodiversity and environmental services. The CCC can help design, implement, monitor and assess reforestation projects, select appropriate livelihood activities and types of reforestation, select communities suited to a reforestation project, guide implementation and understand projects' successes and failure. The CCC also provides a framework to engage with policy makers and funding bodies to explore the types of support for communities to reforest successfully.

This article is part of the theme issue ‘Understanding forest landscape restoration: reinforcing scientific foundations for the UN Decade on Ecosystem Restoration’.

## 1. Background

Reforestation is the intentional process of returning trees to areas from which they were previously cleared. Reforestation can take many forms and have many objectives. It ranges from establishing plantations of fast-growing exotic tree species for timber production or agroforestry systems for diverse crop and tree products through attempts to assist the recovery of the original forest via planting native tree species or enabling natural regeneration [1,2]. Reforestation initiatives, and other conservation and development interventions, that consider local contexts can more effectively address social values and diverse practices that support a variety of livelihoods and promote regenerative economic development and long-term ecosystem stewardship [3–5].

In recent years, there have been substantial international commitments to reforestation, particularly in the tropics. For example, the Bonn Challenge is a global aspiration to restore 350 million hectares by 2030 using Forest and Landscape Reforestation (FLR) as one of the mechanisms to achieve this goal. FLR is an approach under the nature-based solutions movement [6] that aims to enhance tree cover in its amount and quality in an enduring, cost-effective manner for multiple benefits [7]. FLR is one of the many approaches for restoration and builds on social and environmental particularities to integrate objectives and land uses across landscapes [8–10]. Embedded within FLR is recognition of the need to address the livelihoods of rural poor as an integral part of the process [11].

Reforestation and livelihoods are intimately linked [12–14]. Reforestation has the potential to improve smallholder and community livelihoods, particularly in tropical developing countries. Evidence of the ways smallholder livelihoods influence and are influenced by reforestation has been increasingly recognized in the literature since the 1980s [13]. Despite international, national and local efforts, global stocks of natural capital (specifically forests) are in decline. Financial, human, social and physical capitals and other types of natural capital (e.g. increased soil fertility with the use of soil conservation practices) can be brought to bear to build a foundation of natural capital that provides multiple benefits, including increases in global environmental values.

In developing countries, most reforestation initiatives, irrespective of scale, are implemented through the involvement of communities, groups of smallholders and individuals [15], although there are also cases in which reforestation exclude local communities. Communities are highly diverse and have vastly different capacities to implement reforestation activities. In the tropics, there is also a multitude of factors that affect the success or failure of reforestation (e.g. [1,16]) many of which are also related to characteristics of the implementing community [5,17]. Without due consideration of the goals and needs of local communities, there is a great risk of restoration failure [18]. If reforestation is implemented badly, many of the longer term benefits will not be realized, and it may result in detrimental impacts on local communities (e.g. loss of access to land, community conflict, redistribution of wealth to elites). By neglecting the social and political dimensions of restoration, all other benefits (e.g. biodiversity conservation, climate change mitigation and sustainability) can be seriously compromised.

**Figure 1.**
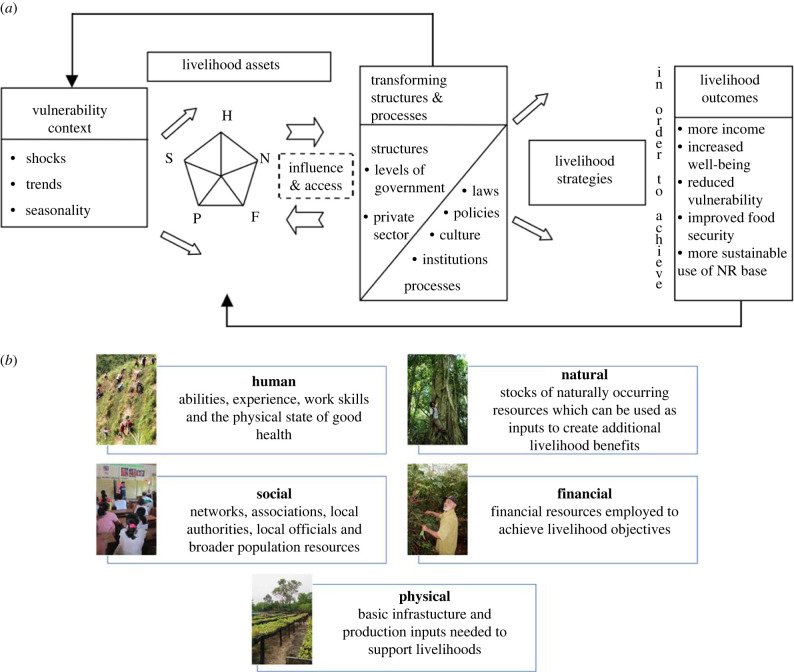
(*a*) The SLF based on five capitals that support livelihoods, source: DFID [19]; (*b*) representation of five capital asset categories. All capitals are important for building community capacity. (Online version in colour.)

Every community and smallholder has a suite of ‘assets’ that they can use for their livelihoods. These assets have been categorized and conceptualized in various ways, including the Sustainable Livelihoods Framework (SLF, [19], figure 1) and the Community Capitals Framework (CCF) [20,21]. The nature and quantum of livelihood assets (figure 1, table 1) possessed by smallholders and communities substantially affect their ability and willingness to become involved in reforestation projects, such as those implemented within the broader context of FLR.

**Table 1.** Livelihood assets definitions and examples relevant to reforestation.

livelihood asset/ capital	definition	references	relevant examples
SLF capital assets			
financial	Financial capital includes financial resources in the form of cash or equivalent, savings, flows and stocks that contribute to consumption and production.	DFID [19] http://www.ennonline.net/dfidsustainableliving (accessed 05/01/2021)	savings; access to credit; income
human	Human capital encompasses a range of attributes, competencies, skills and knowledge held by individuals or groups that enhances their wellbeing. It also includes availability of labour.	OECD [22]	education; skills; entrepreneurship; ability to labour; good health
natural	Natural capital refers to natural resources and services that benefit humans.	DFID [19] (as above)	trees; land; erosion, flood and fire protection; nutrient cycling; biodiversity
physical	Physical capital comprises infrastructure, technology and tools that support livelihoods. It is also referred to as built or produced capital (made by humans).	Ahmed *et al*. [23]	roads; means of communication; technology; equipment
social	Social capital is commonly defined as social structures, relationships and networks. However, some scholars argue for a more comprehensive definition of the term to include the quality of these relationships (e.g. level of trust and reciprocity) and/or institutional tools within these structures and networks (e.g. regulations).	Szreter and Woolcock [24]	participation in community groups; good relationship between community organization and government agencies; shared or synergetic goals and motivations; equitable and inclusive governance structures
	Social capital is also largely discussed in terms of bonding, bridging and linking social capitals. Bonding capital refers to social capital within a group, while bridging and linking refer to social capital across groups, or vertical connections (e.g. to government agencies or external organizations). Bridging capital is related to the social capital between groups of similar levels of power and status (horizontal connections), while linking capital connects people with differential levels of power and status (vertical connections).	Woolcock [25]	
CCF (additional capitals)			
cultural	Cultural capital overlaps with human capital and processes related to institutions and culture. They refer to ‘rules of society’ and factors that provide an inclination for a group to behave in a certain way.	Cochrane [26]	values and needs; preferences; ethics; traditional ecological knowledge; individual and collective histories
political	Political capital overlaps with linking social capital and transforming structures and processes from the SLF. It refers to access to power and connections to organizations that can provide access to resources. It also includes the ability for people and groups to voice their views.	Green and Haines [27]	vertical networks; governance spaces clear rights and responsibilities allocations
		Emery and Flora [28]	

Conceptual models and frameworks based on community capacity and capital assets are available for biodiversity conservation [29], natural resource management (e.g. oyster aquaculture: [30]; water systems: [31]; general natural resources: [32]), tourism [33,34], sustainable development [35] and urbanization [36]. These models demonstrate that community capacity is a complex construct, and that the importance of each capital asset varies with the activity to which it is related, the setting (e.g. urban or rural), and the point in time (i.e. along the implementation continuum of a programme). Community capacity for biodiversity conservation in Australia and natural resource management in Illinois depended less on natural capital, while social, human and financial capitals (e.g. organizations, skills and funding, respectively) were found highly important [29,32]. Natural capital was, on the other hand, crucial for the capacity of First Nations communities for tourism development in Canada [33]. Physical capital was deemed important in conservation in Illinois but was not critical in Australia. And in Australia, the types of knowledge needed for conservation (i.e. human capital) in rural and urban settings differed. The models also demonstrated that community capacity influences what a collective group of people is likely to achieve. For instance, Himes-Cornell *et al*. [37] used the CCF in Alaska, articulated around two major events (i.e. an earthquake and an oil spill incident), to understand how community characteristics influence their preparedness, response to, and recovery from successive or multiple disasters. Additionally, in Illinois, group capacity was predictive of the degree of natural resource management plan implementation success [32].

This paper explores the relationship between communities' livelihood assets and the level of reforestation success. The level of success is defined as the degree to which reforestation objectives at different points in time and different scales are met. Our definition of success also includes the notion that objectives of different stakeholder groups are either synergetic or negotiated. Mismatches between goals of project managers and local land users, for instance, can lead to conflicts and project failure [18].

Here we first develop a conceptual model to characterize community capacity in relation to reforestation. We use the model to illustrate that communities with various levels and types of livelihood assets require different amounts and types of assistance if reforestation projects are to achieve a full scope of socioenvironmental objectives. We outline how this conceptual model can be used to guide the design, development, implementation and monitoring of reforestation programmes and individual projects to communicate on changes realized, to engage with policy makers and inform policy decisions. The assumptions made in the development of the conceptual model include (i) at least a sub-group of the community is interested in reforestation and there is no strong antagonism to it; (ii) communities have suitable land available for reforestation; (iii) communities are implementers of reforestation and benefit from it and are not only considered to be hired labour or contractors; and (iv) communities have clear land or land-use rights so that they can become recipients of reforestation benefits or rights can be obtained in the process.

Communities and other groups of local stakeholders do not exist in vacuums: their decisions and activities are shaped by multiple external factors (e.g. markets dynamics and legal frameworks) that together constitute complex socioenvironmental systems that can enable or hinder their ability to achieve desired goals. Moreover, communities are heterogeneous among themselves and are comprised members with diverse histories, sets of skills, motivations, objectives and perspectives. Not all external factors that affect community capacity and reforestation were included in this study. The relationship between livelihood assets and reforestation is representative of our collective experiences in a range of conservation and development interventions across a variety of tropical countries, particularly in the Asia-Pacific region but also Africa and Latin America. In particular, our conceptual model is strongly influenced by our experiences in implementing a pilot reforestation programme with a single community in the Philippines followed by the scaling out of this initiative to nine additional Filipino communities in three regions of the country.

## 2. Livelihood assets

In conceptualizing the link between reforestation success and community capacity, we draw on the SLF developed by the UK's Department for International Development (DFID). The SLF was one of the first livelihood approaches developed to help design, monitor and evaluate interventions for poverty alleviation. The framework comprises the vulnerability context, livelihood assets, transforming structures and processes (or policies, institutions, and processes in later versions of the framework), strategies and outcomes, and the relationships among them (figure 1*a*,*b*). The five classes of livelihood assets are broadly described in table 1. The SLF approach considers livelihoods to encompass the resources, capacities and activities needed to live/subsist [38].

Of several other theoretical frameworks for characterizing and assessing livelihoods, the most relevant is the CCF (see [20,21]). The CCF provides a way to organize information and ideas about how community development takes place, as participants in community leadership development efforts leverage the resources represented by the CCF among and against one another. The CCF has been proposed as a method for understanding the nature of and processes underlying community development [39].

There are a few important distinctions between the CCF and the SLF. From the perspective of conceptualizing the relations between reforestation and community capacity, one of the deficiencies of the CCF is that it focuses solely on capitals and not on processes. By contrast, the SLF explicitly places livelihood assets in the contexts of transforming structures and processes, including laws and policies. The CCF accounts for these factors through the two additional ‘external’ (i.e. beyond the direct control of the community) capitals, namely political and cultural. As such, the seven CCF capitals include both internal and external factors within the capitals whereas the five SLF assets are identified separately in the ‘transforming structures and processes’ (figure 1). The other main difference between these two frameworks is that the unit of analysis of the CCF is the community whereas the SLF focuses on the individual. In this paper, some components of the CCF are used to complement the SLF. We exemplify the conceptual framework with cases at the community level, although the framework could also be applied at the level of individual landholder or group of landholders. Further discussion of the similarities and differences between the two theoretical frameworks can be found in Guitierrez-Montes *et al.* [38].

In our conceptualization of the relationship between community capacity and the likelihood of reforestation success, we interchangeably use the terms ‘asset’ as per the SLF and ‘capital’ as per the CCF. These two terms, while having slightly different conceptual underpinnings, have the same practical applications for our purposes, i.e. as a means of articulating the collective capacity of a community to undertake reforestation, including the ability to implement reforestation projects, gain additional resources and allocate those resources for community benefit. Community assets/capitals research can provide key insights into the assessment of the capacity a community has to undertake reforestation, regardless of the conceptual model applied.

## 3. Livelihood assets and reforestation

All five categories of capital assets (financial, human, natural, physical and social) can affect implementation of reforestation by communities [14]. Below we outline the five capitals from the SLF and the two additional capitals (i.e. political and cultural) used in other frameworks and show how each relates to reforestation.

### (a) Financial assets

Communities need financial assets to embark on reforestation. Reforestation can be a high-risk activity with high upfront costs and benefits that are not received until many years in the future [40]. Communities will need to address cash flow needs during this time gap. The lack of funds to finance projects of this nature is a major impediment to reforestation (e.g. in Vietnam [41] and in the Philippines [42]). Often borrowing money is not an option due to limited motivation for financial institutions to provide such services [43,44] due to factors such as insecure land tenure of borrowers [45,46], and the lack of the forms of collateral generally required by banks [47]. Nevertheless, there are examples where micro-financing enabled reforestation (e.g. [48]). Typically, communities and smallholders must either self-finance reforestation or be provided with some form of financial support such as grants or short-term income streams from associated livelihood projects (e.g. payments to grow seedlings or plant trees). Direct payment for such services may often be the main motivation for people to engage in reforestation activities. Other options to boost financial capital include payments for environmental services and advanced payments for timber or carbon sequestration. Typically, financial capital is most critical for the poorest communities. Apart from financial assets that directly enable reforestation success, the characteristics of the sources of income of a community is also important. The regularity, source (i.e. on-farm or off-farm), amount of income, type and amount of foregone revenue of alternative land uses, and the financial risks that exist and differ even within communities also define a community's willingness and ability to devote labour and land for reforestation. Also, community members will have different risk profiles. More well-off individuals may be able to take on more risk, or at least may be more willing and able to invest in reforestation initiatives.

### (b) Human assets

Reforestation can be labour intensive at the start and through early stages of implementation for the establishment activities and maintenance, particularly when trees are young, and weeds have not been suppressed yet. Labour availability may likely depend on wage subsidies that equal or exceed wages available from other sources. Skills and knowledge of reforestation implementers are also important [1,14,49]. Low levels of silvicultural knowledge can hamper tree planting efforts and affect final tree quality [50–52]. However, low levels of these assets in a community can be addressed if social capital is high and there is access to information and training (e.g. [53]), particularly where the opportunity cost of labour is low.

### (c) Natural assets

To plant trees, communities need access to suitable land. Typically, this land is marginal for agriculture, often degraded and of difficult access [14]. Under these conditions, the opportunity costs of restoration are low, but the implementation costs are high. Additionally, marginal land may be unsuitable for certain types of reforestation, especially if timber production is an objective [54–56]. Land scarcity is a major constraint on reforestation. Other types of natural capital important for reforestation include availability of adequate plant material [5,57] and the ready availability of water. The level of natural capital for a given community can be linked to their motivation for landscape restoration. Where a community is more vulnerable to negative impacts from losses of natural capital, they are more likely to be motivated towards restoration activities. The type and quality of natural assets available may also influence types of reforestation required. For instance, in communities where harvests reduced the abundance of useful species, there may be a focus on replenishing the forest with such species. In other settings, restoration activities will be more linked to agroforestry so that access to subsistence agricultural products is not threatened.

### (d) Physical assets

Communities need access to infrastructure and technology to successfully grow trees [51,56,58]. Requirements may include nurseries to raise seedlings and access to clean water to the community through to more basic planting implements such as shovels and watering cans. Roads and fencing materials, access to planting or assisted natural regeneration sites, and vehicles can also have a large impact on the maintenance and protection of reforested areas. Availability of physical assets also influences the potential to produce, harvest and process forest products and to access markets [59–61] and for promoting information exchanges with other key actors [41].

### (e) Social assets

Social assets are essential for successful reforestation. The levels of bridging, bonding and linking social capital (defined in table 1) affect the success of reforestation [17,62]. In addition to participation in community groups, group cohesion, shared vision and synergetic goals, ability to negotiate, existence of trust in relationships within and beyond communities, equity within communities and promotion of equitable practices, available connections to external groups and agencies for exchange of resources, and effective communication platforms are also important, among many other factors.

### (f) Cultural assets

Cultural assets overlap with human and social capital and processes related to institutions and culture (figure 1). Traditional ecological knowledge can play essential roles in reforestation activities. Shared cultural and religious values that promote the protection of forest and forest resources often encourage reforestation directly and indirectly. Certain locations, plants or practices that have cultural significance can provide a strong motivation for the undertaking of reforestation. Indirectly, shared cultural and religious values can enhance community cohesion and collective work. However, in cases where areas being reforested have been recently colonized, such as in some upland areas in the Philippines opened up to in-migrants by the construction of logging roads, traditional knowledge may be scarce.

### (g) Political assets

Political assets overlap with social capital and transforming structures and policies. Policies, legislation (i.e. allocation of rights and responsibilities), governance arrangements (i.e. legitimacy, transparency and representation), institutions and political support that facilitate reforestation are essential for the transformation of capital assets into reforestation gains. Equitable and inclusive governance processes are among the most important assets for assuring the long-lasting impacts of reforestation.

Some assets cut across multiple capital asset categories. Land tenure, for example, falls under most of the categories depending on the perspective taken. Land tenure security (either formal or informal) is a key factor influencing community and smallholder engagement in tree planting and long-term maintenance and protection of reforested areas (see [17,63–65]). If viewed as an institutional instrument that defines rights and responsibilities, land tenure is a form of political capital. To the extent that it unites community members and empowers their collective sense vis-à-vis society, it represents a form of social capital. If land tenure is perceived as a means of production or access to land, it represents an element of physical or natural capitals.

#### (i) Capacity for communities to undertake reforestation

Community capacity can be defined as the collective ability of a group to identify, organize and address issues and opportunities by mobilizing various forms of capital within institutional and relational contexts [66]. Beckley *et al*. [67] proposed a model of community capacity, based on research in rural Canada, involving four elements—capital assets, catalysts, social relations and type of outcomes. According to these authors, community capacity is influenced by the nature of the catalyst for change, because community motivation and capacity to address different issues and opportunities vary. Motivation will be influenced by types of outcomes sought from reforestation. For instance, at the policy, programme and project scale, macro-level outcomes, such as increased tree cover, may be sought, whereas communities and families may be motivated by micro-level outcomes such as specific livelihood or environmental benefits of individual activities or plantings. Alignment of macro-level with specific community-supported reforestation outcomes can influence motivation and community capacity.

The capacity of communities to undertake reforestation is represented by the collection of assets they can access and use. Social and human assets can be improved through capacity building interventions that mobilize existing capital to achieve the desired objectives [68]. Therefore, the capabilities of communities to undertake reforestation can be improved with strategically targeted capacity building on topics such as financial management, leadership and technical knowledge. Reforestation also changes livelihood assets. If reforestation is implemented well, these changes are likely to be mostly positive, including increases in income, improved environmental conditions, greater skills of community members and an improved ability of communities to access external support. Other non-tangible benefits include community consolidation and enhancement of a sense of ownership. Reforestation can also have deleterious impacts such as loss of autonomy, reductions in access to arable land and associated reductions in incomes and food security, or increased labour requirements that impose additional and/or inequitable burdens on community members.

Greater community capacity (i.e. combined community livelihood assets) increases the likelihood that reforestation projects will succeed in delivering broad socioenvironmental outcomes. It has become clear that the ability of communities to undertake reforestation is related to the various assets that a community can use to establish and maintain trees (see [14]). This could be a simple linear relationship whereby the greater quantum of assets a community possess the greater likelihood of reforestation success. By contrast, the relationship might be better represented by some other curvilinear form such as an exponential, logarithmic or sigmoidal function. An exponential relationship would indicate that relatively small improvements in capital stocks result in substantial increases in the likelihood of success. By contrast, the opposite would be the case for a logarithmic relationship whereby an increasingly higher quantum of livelihood assets is required to produce a relatively small incremental change in the likelihood of reforestation success. A sigmoidal function implies there are two tipping points. On the first one, there is an exponential increase in the likelihood of success with small incremental changes in capacity. On the second one the likelihood of success has small incremental gains despite the increase in capacity.

## 4. The community capacity curve applied to reforestation

**Figure 2.**
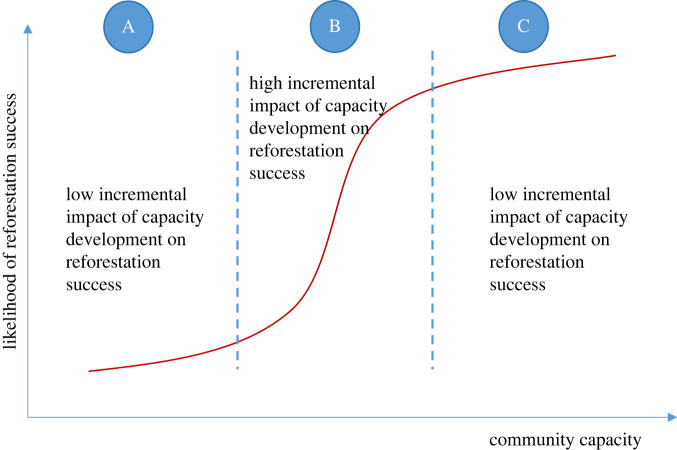
The CCC applied to reforestation and the varying impacts of capacity development interventions on the likelihood of reforestation success. (Online version in colour.)

We argue that a sigmoidal function most faithfully describes the relationship between community capacity and reforestation success (figure 2). There is likely to be a certain base level of capacity required for reforestation to be successful—communities need some minimal threshold of financial resources, social capital, access to land, technical skills and equipment, etc. Below this level, reforestation success is unlikely. Gains from reforestation when working with communities at the lower end of the curve can be very significant, both in the environmental and socioeconomic spheres. However, for these gains to be achieved, capacity limitations must be overcome, and the position of a community on the curve must be moved to the left, i.e. up the curve. Because communities differ in the extent to which they have these assets, they differ in their asset needs. In the initial, shallow stage of the curve, investments in improving the various capitals are unlikely to greatly improve the likely success of reforestation projects by communities (including the initial implementation of projects) as there is a minimum level of each capital required for success and these minimum levels of capitals are likely to vary across geographical and socio-political contexts (figure 2, stage A). As community capacity increases, the steeper mid-stage of the curve is reached where modest investments in improving community capacity are likely to result in much greater increases in the likelihood of reforestation projects being successful (figure 2, stage B). At some point, this high rate of increase in likelihood of success is expected to tail off, for example, for communities located in the upper shallow section of the community capacity curve (CCC) (figure 2, stage C).

There are some fundamental questions as to how to represent the stocks of capitals, whether all capitals are of equal importance across all sectors of the curve and their degree of interchangeability, complementarity or trade-offs. These issues are discussed below and illustrated with examples.

## 5. Placing communities on the curve

Several options exist for identifying where communities are placed on the curve. For example, the classification of communities at different stages of the curve can be based on qualitative assessments based on expert judgement by those knowledgeable about the communities. Another means to identifying the level of community capacity is by using mixed methods approaches based on information gathered directly from communities.

People who work with communities (e.g. government officials, NGOs and researchers) often understand their capacities to undertake projects. When we discussed the CCC model (figure 2) with this type of practitioners, we regularly found that they intuitively and easily place the communities with which they work on the curve in relation to other communities with which they are familiar. This result is consistent for practitioners working with communities from a broad range of locations including for indigenous communities in northern Australia, the Philippines, Vanuatu, Papua New Guinea and Brazil. The precise assigned position often varies a bit, but most experienced practitioners have been very comfortable placing communities within a general area along the curve.

**Figure 3.**
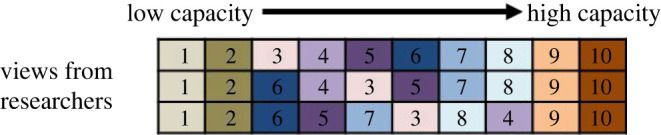
Ranking of 10 communities (one colour each, one number each) in the Philippines regarding their level of capacity (left to right) by three researchers (rows). (Online version in colour.)

To test whether there is a common understanding of community capacity, in a related study conducted by Ota *et al.* ([69], in preparation), three researchers with knowledge about 10 communities that participate in a forest restoration project in the Philippines were asked to rank these communities with respect to their ‘performance’ in reforestation. The researchers were familiar with the SLF framework. In this case, performance was not measured using common metrics such as seedling survival rate because the project provided sufficient support for each community to achieve high seedling survival rates. Instead, performance was assessed based on the support required to achieve community goals, the inputs provided by the communities, and their level of preparedness for reforestation. From this exercise, the position of each community on the curve was approximated albeit with no explicit consideration of the distance between them. There was an agreement on the ranking of the 10 communities between the researchers with a Kendall's W coefficient of 0.88 ([69], figure 3).

**Figure 4.**
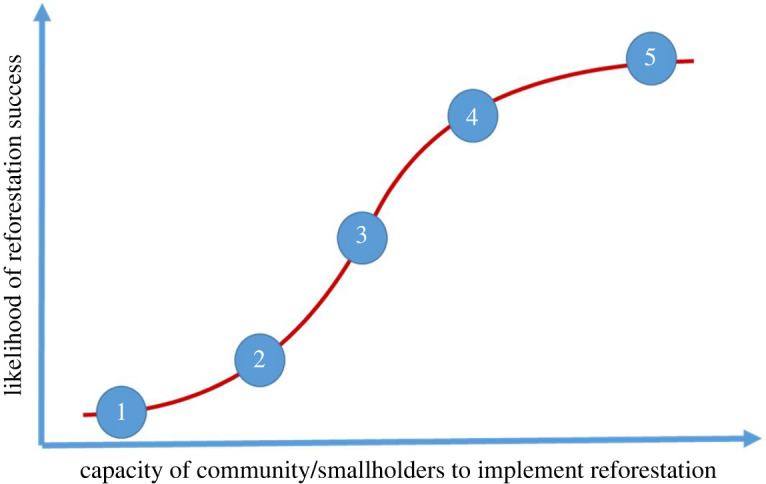
Positions of archetypal communities on the CCC applied to reforestation. (Online version in colour.)

Another way to locate communities along the curve is to apply a landholder typology approach (e.g. see [70,71]) that could comprise five archetypes (categories) of communities (figure 4), and these archetypes are described by their typical characteristics based on the five capitals. Examples of characteristics related to capacity that could be used in the development of typologies include attitudes towards risk, income, land size, availability of labour, land use and motivations, among others. Using this information, the placement of communities on the curve could be estimated, for example, between 1 and 2, just above 3, etc. (figure 4). Information from the typologies can be used to identify what would need to change to move communities from one point to another, and the enabling and limiting factors that would underlie these potential transitions.

**Figure 5.**
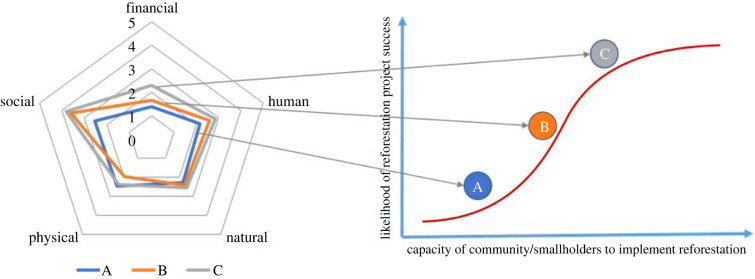
Average Likert scale scores per capital category per group related to the position in the CCC (source: [69]). (Online version in colour.)

Several quantitative approaches could also be used to identify where communities are located on the curve. For instance, Likert scale measures of key elements of the five capitals could be obtained through surveys and observation. A metric could then be developed or spider diagrams of each of the five capitals could be used to visually display quantification of capitals using the Likert scales (figure 5).

Figure 5 follows on from the ranking of communities presented in figure 3 in relation to their performance in reforestation. It uses Likert scales on 41 variables related to the five capital assets from the 10 communities mentioned previously. The spider diagram provides a good means to communicate community capacity, despite the fact that the area of the shapes is not directly related to the level of capacity of a community (i.e. the area of the shape increases as a square of volumes rather than linearly and the order of the categories impacts shape). A more quantitative analytical approach could also be used to estimate the position of communities on the curve. In a non-metric multi-dimensional scaling analysis (stress = 0.136, 20 runs, 2 dimensions) with the 41 variables, both axes correlated closely with position of the communities in the curve. Communities at the bottom, middle and top of the curve as classified by experts in regard to their performance in reforestation differed significantly, validating the method of classification by experts in this case. The variables that caused the difference between the three groups were diverse and related to all five capital assets assessed (i.e. financial, human, natural, social and physical capitals), as identified in a SIMPER analysis of dissimilarity between the groups [69].

### (a) Applications of the community capacity curve

We contend that the CCC has many potential applications for the design, planning, implementation, monitoring and assessment of reforestation projects with communities, including national and international policies related to reforestation (e.g. FLR). In addition, it can be used to evaluate what types of interventions and capacity development are needed in particular cases, and when analysed along with external factors, to explain the reason behind the failure of certain projects.

#### (i) Planning and design of reforestation projects

As part of any reforestation project, an initial assessment of community capacity can help identify strategic immediate actions and resources to ensure that the necessary inputs are made available to ensure communities have both the short- and long-term capacity required for the objectives to be met. As outlined previously, communities in stage A (figure 2) are resource-poor with little capital and are thus more likely to depend on more immediate livelihood benefits. That is not to say that communities in stage A are not environmentally concerned and do not have aims related to ecosystem restoration, but rather that livelihood needs are of more immediate importance. Stage A communities also require high levels of support, including skills-building, and typically depend on direct cash payments for their continued involvement at least until they secure the benefits of alternative subsistence activities (e.g. small-scale agriculture). Communities in stage C, in contrast, with more capital, are more likely to not require livelihood projects and have greater ability to focus on restoring ecosystem goods and services (e.g. by planting native tree species). Projects involving elements of biodiversity conservation, including those focussed on restoring habitat or endangered species are more likely to succeed when implemented by communities that have already graduated to stage C of the curve.

#### (ii) Selection of livelihood activities and types of reforestation

**Figure 6.**
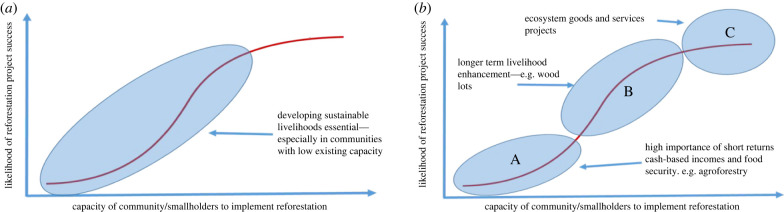
(*a*) Communities that most require sustainable livelihoods and (*b*) types of sustainable livelihoods that vary with position on capacity curve. (Online version in colour.)

There are many different ways in which reforestation can be achieved including boundary plantings, agroforestry, small monoculture woodlots, mixed species woodlots, high-diversity reforestation plantings and assisted natural regeneration. Similarly, countless livelihood activities can support engagement of communities and individuals in reforestation. The position of a community on the curve will influence the type of reforestation that is most appropriate, the type of livelihood support that is required, the likely level of stakeholder engagement, and the ability of the community to partner with other groups. These factors, in turn, influence the socioenvironmental outcomes that can be achieved. Most communities in developing countries fall within the lower to middle position of the CCC (figure 6*a*). This means that for most, the development of sustainable livelihoods should be an essential part of almost all reforestation projects. Communities at the lower end of the capacity curve (figure 6*b*) have few existing livelihood options, low-financial capital and technical skills and lack resources that can be directed to reforestation, although other useful resources might be abundant (e.g. traditional knowledge, labour availability). Achieving food security and short-term returns from cash-based incomes in these communities is likely to be of highest priority if the goal is for these communities to engage in reforestation activities. Fast-growing species that provide multiple products, and especially those that allow early harvest of non-timber products such as firewood, along with agroforestry-based systems are most suited to communities that sit at the lower part of the curve. Non-forest-based livelihoods providing immediate returns such as livestock husbandry, handicrafts, apiculture and vermicomposting are also preferred by communities at the bottom of the curve and consideration should be given to implementing them as part of reforestation projects. These forestry/agroforestry systems help satisfy the basic shortfalls in financial capital that are characteristic of communities with low capacity. Assisted natural regeneration might be another suitable option due to its low cost, if the environmental conditions allow (see [72]).

Communities further along the capacity curve have less immediate need for cash and subsistence outcomes and have a greater capacity to invest in longer term benefits. Planting of fruit trees (e.g. cacao and coffee) and high-value timber species that yield income after substantial delays require greater human capital (e.g. knowledge of how to manage), financial capital (e.g. costly planting stock), social capital (e.g. ability to access training, market chains, etc.) and physical capital (e.g. processing and transport facilities). For instance, livelihood projects with delayed cash flows such as fruit trees, cacao and coffee, might be more suitable for communities in stages B or C of the capacity curve, characterized by communities that have more secure forms of livelihood income.

Communities at the top are not overly reliant on the plantings to support their immediate livelihoods; for example, they might have off-farm employment or not live on farm areas. These communities are most likely to have capacity to implement projects that provide longer term environmental benefits and are more likely to consider payments for environmental services as supplementary. These communities are also the most likely to be able to implement forest restoration projects that involve diverse plantings of native species, at least if the incentives for doing so are available.

#### (iii) Assisting communities to be ready for restoration

Understanding where a community sits on the capacity curve at the start of a restoration project and as the project develops has important equity implications. If a project or programme focuses on restoring biodiversity using native species or providing ecosystem services with limited or delayed compensation of the landholders involved, then these types of projects are only suitable for communities at the upper end of the curve. It is unrealistic and unfair to expect low-capacity communities to be interested in implementing these types of projects when their focus is primarily on subsistence and basic livelihood activities. To include communities at the lower end of the curve in such projects, NGOs and funding agencies must be prepared to provide substantial capacity building and short-term livelihood projects and be committed to providing support over many years, until the trees planted start to produce commercial products. This intensive support must be accompanied by appropriate institutional choices related to which partners to involve in the project, stakeholder groups' rights and responsibilities, building trust in relationships among stakeholder groups, having local people and communities involved at all phases of the project, and having staff that can navigate complex socioecological systems [73]. Determining a community's position on the CCC might provide an indication of where and when payments for environmental services can effectively promote forest restoration.

#### (iv) Implementing reforestation projects and understanding project successes and failures—a case study from Biliran

**Figure 7.**
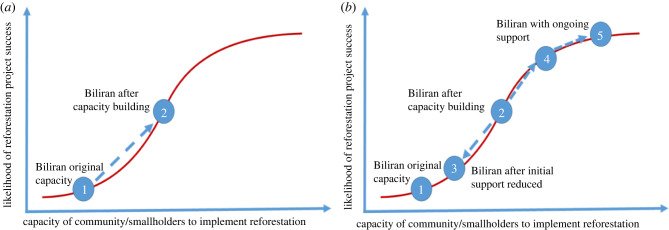
Changes in capacity of Biliran community to undertake successful reforestation (*a*) after initial interventions and (*b*) after withdrawal of cash support and then further assistance. (Online version in colour.)

Many of our ideas about community capacity were refined as part of an ongoing pilot testing of reforestation best practices with a community in Biliran, Philippines (details can be found in the electronic supplementary material, Appendix). As part of this project's implementation, we spent about 12 months building capacity of the community to undertake reforestation. Activities focused on building of social capital (e.g. training in governance procedures, assisting to reform disbanded communities, resolving existing conflicts, promoting practices that enhance the influence of marginal groups); provision of physical capital (e.g. tree nursery); financial capital (payments for tree planting, financial support to establish an agroforestry farm); and human capital (technical training in nursery techniques and silviculture). The various capacity-building activities pushed the community up the reforestation curve (figure 7*a*, point 1 to point 2) in about 2 years. The initial participation of community members was driven by short-term financial incentives, especially cash payments for production of seedlings and the subsequent planting of these seedlings.

After about 3 years we started to withdraw support, especially the cash payments, with the expectation that the reforestation activities would be sustained with only advisory support. We found that the capacity of the community to continue with reforestation was limited, largely because community members sought additional paid work to replace the income lost when cash payments ceased for nursery and planting activities. Because men had more opportunities for paid outside work, the burden of reforestation-related activities fell mostly on women. It was clear that the financial capital we provided was fundamental to the initial success envisioned for this stage—and raised the overall capacity of the community to a level at which reforestation successes were achieved. However, once this financial capital input ceased, the overall community capacity to continue implementing reforestation activities dropped along the curve and the reforestation project was close to failure (i.e. maintenance and protection of planted trees decreased to the point that high mortality was likely if further support was not provided). This is represented in figure 7*b*, point 2 to point 3. In discussion with the community, project managers then decided that further intervention was required and provided additional financial and technical support for another year. This support pushed the community further up the curve—estimated at point 4. To this day, the community has continued to improve its capacity to undertake reforestation. Improvements in social capital, especially linking social capital (defined in table 1), have resulted in the community successfully applying for financial support from the Philippines Department of Agriculture to establish additional agroforestry areas. In addition, the community was awarded funding from the Department of Environment and Natural Resources to reforest an additional 60 ha. This result suggests that the community is on an upward trajectory (e.g. moving towards point 4 or 5, figure 7*b*) where reforestation efforts are likely to be sustained. The long-term commitment to managing the trees can be attributed mainly to development of sustainable livelihoods, securing land and tree rights, agreeing on mechanisms for equitable sharing of benefits, strong leadership, effective governance and improvement of other components of human and social capitals [74].

### (b) Capital substitution: are all capitals the same, how much is needed of each and how much can they be substituted?

Can deficiencies in one or more capitals be counterbalanced by increasing other capitals? For example, providing financial capital through cash payments to plant trees might compensate for lack of social or physical capital. The extensive review of the factors affecting the success of community forestry groups by Baynes *et al*. [17] suggests that some assets are likely to be more important than others, namely socioeconomic status (financial capital), secure property rights (physical capital), good intra-community governance (social capital) and material benefits (financial capital) along with government support (financial capital) at some and possibly all points in time along the implementation of a project. The seven initial restoration flagship cases collected by the Crowther Lab (https://crowtherlab.com/flagship-cases/) demonstrate that social capital and improving it are important enabling and success factors for restoration both in community-based initiatives and those led by individuals and organizations. For example, a community-based effort initiated by the non-government organization DECOIN in Ecuador was able to achieve outcomes that would be unattainable by individuals and was considered to encourage more inclusive participation and knowledge sharing [75]. The community had a history of working together and a high level of cohesion. In other examples, in private and individual-based restoration in Brazil [76], Lebanon [77] and Tanzania [78], coordination among stakeholders and creating spaces for exchange of experiences and knowledge were deemed important factors for the progress of restoration. In addition, social capital allows a greater ability of communities to secure and at later stages, defend property rights, obtain material benefits and share these equitably, and to access government support.

The importance of each type of asset is highly context specific (e.g. policy environment, types of pressure on the landscape). Our experiences with the Biliran community also suggest that not all capitals are equal and that they are not necessarily substitutable. The cash support provided to the community boosted its overall capacity to a point where reforestation became feasible. However, the impact of this financial subsidy was temporary and the community's capacity to continue with reforestation quickly diminished once this support ended. With further development of social capital and given the already lived experiences, the community was able to source additional support from other sources. The building of social capital within communities can be complex and is influenced by historical context. In communities burdened by negative social issues (e.g. [79]), social capital building activities (i.e. promoting transparent governance structures and disbanding existing corrupt groups) and/or the introduction of financial capital can potentially undermine bonding social capital.

These experiences have two important implications. First, external financial subsidies, while critical, typically build transient or temporary financial capacity. Once financial support ceases, the capacity it represents quickly disappears. This outcome contrasts with social capital development, which builds more permanent capacity [80]. The gains in social capital catalysed a process of capital transformation, which enhances the likelihood of continued reforestation. Second, it is clear that financial and social capitals are not directly substitutable, at least in the terms of prospects for longer term success of reforestation. The widespread failure of reforestation projects in the Philippines and elsewhere in the tropics after financial support is withdrawn provides strong support for this statement [15]. Simply put, short-term grants and payments to communities for reforestation buys temporary increases in financial capacity to undertake reforestation, but this capacity is not permanent, and when payments stop, communities slip back down the curve as in the example in figure 7*b*.

For the 10 communities in the study by Ota *et al.* [69], financial, social and human capitals diverge most strongly among communities at different stages along the CCC (figure 5, spider diagram). Financial capital is the one that most varies depending on the position along the curve, whereas social capital often appears similar among communities on the middle and upper parts of the curve. The lack of social capital in communities at the lower end of the curve (e.g. Biliran) could be one of the reasons why when financial support is withdrawn these communities then slip back down the curve. In other words, the communities in this case do not have the social and political systems to be able to convert the short-term cash payments into lasting or permanent financial assets. This is also influenced by factors external to the community. The limitation in social capital sometimes is more prominent in terms of linking and bridging social capitals, while in other situations, it also involves limited bonding social capital. This result also lends support to the realization that not all assets are equal in terms of importance to the implementation of successful reforestation projects. Despite the importance of natural capital on which forest restoration is built, this capital asset category was not found decisive in reforestation outcomes [69]. Perhaps this may reflect that for communities to be involved in reforestation projects they must have access to land and thus once this requirement is satisfied, it does not then markedly affect reforestation performance. Limitations in natural capital were found to be easier to overcome than in other capital asset categories in the communities studied in the Philippines. For example, the community with lands with the most challenging planting conditions in terms of soil fertility and depth was also the community with the highest level of social capital and the best performance in reforestation. In this case, social capital had a more defining influence on reforestation performance than soil fertility because reforestation in this case involved tree planting and tending. If the restoration action was based on natural regeneration, environmental conditions might have played a larger role.

## 6. General discussion

We have developed the CCC framework primarily as a means of conceptualizing the relationships between the assets communities have at their disposal to implement reforestation and the likelihood of success. We found it a useful tool to guide our own collaborative community-based research and practice, and to communicate with policy makers and implementers of reforestation about the importance of developing community capacity and how community capacity is as an enabling factor for restoration along with other factors operating at different scales. Importantly, the CCC highlights that all communities are not the same in their ability to reforest, which implies that standardized interventions adopted across landscapes and nations are doomed to fail. It proposes a more equitable approach in which communities with lower capacities are identified to receive higher levels of support.

Capacity development and support to communities for reforestation is almost always needed across a range of domains including technical, social, financial, management, legal, etc. [81,82]. Capacity is also needed to build trust and develop a shared vision among reforestation actors regarding the nature of issues and potential pathways to address them. The CCC framework has a temporal aspect and a dynamic nature. Diverse needs and opportunities exist at different phases of reforestation depending on the level of capacity of a community. The CCC applied to reforestation provides a way to identify these needs and to understand differences among communities, especially in discussions with policy makers and implementers of reforestation projects. Importantly, while we used the framework in the context of communities undertaking reforestation, the general concepts can also be equally applied to other areas of research and development including natural resources management (e.g. fisheries), agriculture and health promotion. Information gathered and processes related to the characterization of community capacity can help build and consolidate community identity and further their understanding of the change process needed to achieve realistic desired goals. In doing so, this framework is a powerful tool to facilitate social learning and community empowerment, to make visible changes made along intervention implementation (i.e. monitoring), and when used in counterfactual settings, to elicit relevant information on which to base additionality considerations regarding the success of specific interventions. This knowledge has become increasingly important for national governments and donors endeavouring to promote positive socioenvironmental outcomes. Also, capacity is not required only at the community level. Reforestation often cuts across multiple governance levels and it is required at all levels, and there is the need for platforms for dialogue between these levels [83]. In this case, the CCC framework becomes a tool to socialize and build collective knowledge and shared understanding of realistic goals to be achieved by particular community actors with respect to restoration goals.

The sigmoidal shape of the CCC reflects our belief that there is a certain minimum amount of capacity required for sustained positive reforestation outcomes. In theory, reforestation projects implemented in communities beyond the initial inflection point are likely to succeed. The communities higher on the curve already have more financial, human and social capital but may lack some specific skills, networks, or knowledge. In such cases, strategic short-term assistance may be sufficient for them to achieve their reforestation goals. Such capacity building may be in the form of training in nursery production techniques, selection of appropriate species, design of reforestation interventions, ability to interact with a specific type of stakeholder (e.g. private sector), etc. An *ex-ante* assessment of the combination of capitals a community possesses (and hence its position on the curve) may also be used as a leading indicator (see [84]) or for baseline assessment by agencies implementing reforestation (e.g. determination of likelihood of reforestation success; assessing the need to provide targeted capacity development). Examples of assets to be evaluated at this stage include ability to address within-community conflicts; transparency in community activities; level of equity in decision making; tenure security; availability of socioeconomic buffers; amount of savings; level of access to main market centres; and technologies available. These can also be assessed at later stages of the process as both leading and lagging indicators as part of a monitoring programme for tracking changes and making adaptive management decisions [84].

Our approach is based on the five capitals identified by SLF. Within that framework, political and cultural issues are captured within the ‘transforming structures and processes’ box of figure 1*a*. Culture is a potential important contributor to the capacity of communities to undertake reforestation. Culture, local ecological knowledge and the historical attachment of a community to the landscape potentially play an important role in the way communities view their roles in reforestation. For instance, in certain parts of the world, communities with a long multi-generational association with a landscape might be more likely to view reforestation in the context of preserving the traditional stewardship of the land and are more likely to be amenable to redirecting community assets to achieving reforestation. By contrast, in-migrants to upland areas made accessible by logging are likely to view the land as a means of producing income rather than as part of their cultural heritage. In these cases, reforestation would be at best seen as an income-generating opportunity through payments to plant trees, and at worst, as a restriction on their livelihood activities.

Reforestation is a long-term process, with multi-year commitments required. There is increasing recognition that many reforestation projects may initially meet their short-term goals (i.e. tree seedling planted and targeted area established) but then ultimately fail to meet their longer term reforestation goals [84]. The failure of community reforestation can be explained in terms of the CCC. For communities at the bottom of the curve, the support provided (i.e. seedlings, payment for labour) pushes the community transitorily up the curve. These forms of support do not address the underlying community capacity required to support reforestation over the long run. In effect, the funding and support by NGOs and donors push communities temporarily up the reforestation curve—which facilitates the community involvement in planting of trees. However, once the support is withdrawn (e.g. financial capital is no longer provided) then the community capacity declines, and the community falls back down the CCC leading to project failure. For reforestation project impacts to be sustainable, community capacity must be boosted to levels past the inflection point on the CCC to avoid sliding back. To reach this point, communities lower down the curve will require sustained and often substantial capacity building and assistance.

As opposed to providing short-term cash payments, developing social capital provides lasting benefits to communities but is often neglected by support agencies, including both governmental and non-governmental organizations. As such, programmes that boost social capital and hence move communities up the CCC are more likely to result in a permanent shift up the curve. This is not to say that only social capital should be enhanced as part of reforestation projects. Poor communities simply cannot afford to implement reforestation without additional financial and physical resources being provided. However, once these resources are made available to communities, lasting increases in social capital make it more likely that communities will be able to replace or seek resources to continue reforestation activities once support ceases, consolidating the gains in natural capital.

## Data Availability

The data are provided in the electronic supplementary material [85].
